# Increased PD-L1 expression in KRAS mutated premalignant human bronchial epithelial cells is enhanced by LKB1 loss and mediated by ERK activation

**DOI:** 10.1186/2051-1426-3-S2-P305

**Published:** 2015-11-04

**Authors:** Mi-Heon Lee, Jane Yanagawa, Rui Li, Tonya C Walser, Kostyantyn Krysan, Gerald Wang, Jonathan W Goldman, Edward B Garon Garon, Gang Zeng, Sherven Sharma, John D Minna, David Carbone, Steven M Dubinett, Jay M Lee

**Affiliations:** 1 UCLA, Los Angeles, CA, USA; 2 UTSW, Dallas, TX, USA; 3 The Ohio State University, Columbus, OH, USA

## Background

PD-1/PD-L1 immune checkpoint pathway mediates tumor evasion from the immune system, and may be associated with poor prognosis in lung cancer. Activating KRAS mutations and LKB1 loss are common mutations in non-small cell lung carcinoma (NSCLC). Patients with mutated KRAS demonstrate less benefit from chemotherapy and resistance to approved targeted therapies. Inactivation of tumor suppressors commonly co-exist with KRAS mutations, and may contribute to tumor progression and treatment response. LKB1 is a tumor suppressor gene commonly inactivated in lung adenocarcinomas. The role of LKB1loss as a prognostic or predictive marker within human KRAS mutant NSCLC is unclear. There have been no therapeutic strategies that effectively target LKB1. Furthermore, the effect of these mutations on immune checkpoint immune regulation is poorly understood. KRAS mutations are known to activate the RAF-MEK-ERK pathway. We hypothesize that KRAS mutations directly regulate the PD-1/PD-L1 pathway and LKB1 loss may increase this effect through ERK activation.

## Methods

Immortalized human bronchial epithelial cells (HBEC) with null vector and shRNA control (HBEC-vector-sh control), HBEC LKB1 knockdown (HBEC-vector-shLKB1), KRAS–mutated (KRAS^v12^) HBEC cells with shRNA control (HBEC-KRAS-sh control), and LKB1 knockdown KRAS–mutated HBEC cells (HBEC-KRAS-shLKB1) were used to assess mRNA and/or surface protein expression levels of PD-L1 by real time-qPCR (RT-qPCR) and flow cytometry. HBEC-vector and HBEC-KRAS cells were treated with MEK (ERK kinase) inhibitor (PD0325901) at 1uM for 24hrs and evaluated for mRNA and surface protein expression of PD-L1.

## Results

PD-L1 mRNA level increased 2.4 fold (HBEC-KRAS-sh control; p < 0.05) and 4 fold (HBEC-KRAS-shLKB1; p < 0.05) vs. HBEC-vector-sh control. Based on mean fluorescence intensity, PD-L1 protein expression was 7.4 (HBEC-KRAS-sh control) and 12.1 (HBEC-KRAS-shLKB1) vs. 3.4 (HBEC-vector-sh control). With ERK inhibition, PD-L1 mRNA levels decreased 88% (HBEC-KRAS-sh control) and 68% (HBEC-KRAS-shLKB1), and PD-L1 surface protein levels were reduced 77% (HBEC-KRAS-sh control) and 64% (HBEC-KRAS-shLKB1). These findings suggest that ERK activation mediates KRAS mutation driven over-expression of PD-L1 mRNA and protein, which is further increased with LKB1 loss. LKB1 loss alone without KRAS mutation did not significantly affect PD-L1 expression (HBEC-vector-shLKB1 vs. HBEC-vector-sh control).

## Conclusions

Here, we demonstrate that elevated PD-L1 expression in premalignant KRAS mutated human bronchial epithelial cells is enhanced by LKB1 loss and mediated by MAPK/ERK pathway activation. Our findings suggest that activating KRAS mutations and LKB1 loss in NSCLC may predict improved therapeutic efficacy to PD-1/PD-L1 inhibition.

